# Supplementary material for: The interactome of intact mitochondria by cross-linking mass spectrometry provides evidence for coexisting respiratory supercomplexes
Source: Mol Cell Proteomics. 2017 Dec 8;17(2):216–32. doi: 10.1074/mcp.RA117.000470 (PMC5795388; doi:10.1074/mcp.RA117.000470)
Supplement: Supplemental Data [file supp_RA117.000470_133922_0_supp_23978_fzffwf.zip › spectra_annotation/mito_spectra_annotation/487-1-3-1-13-1.pdf]

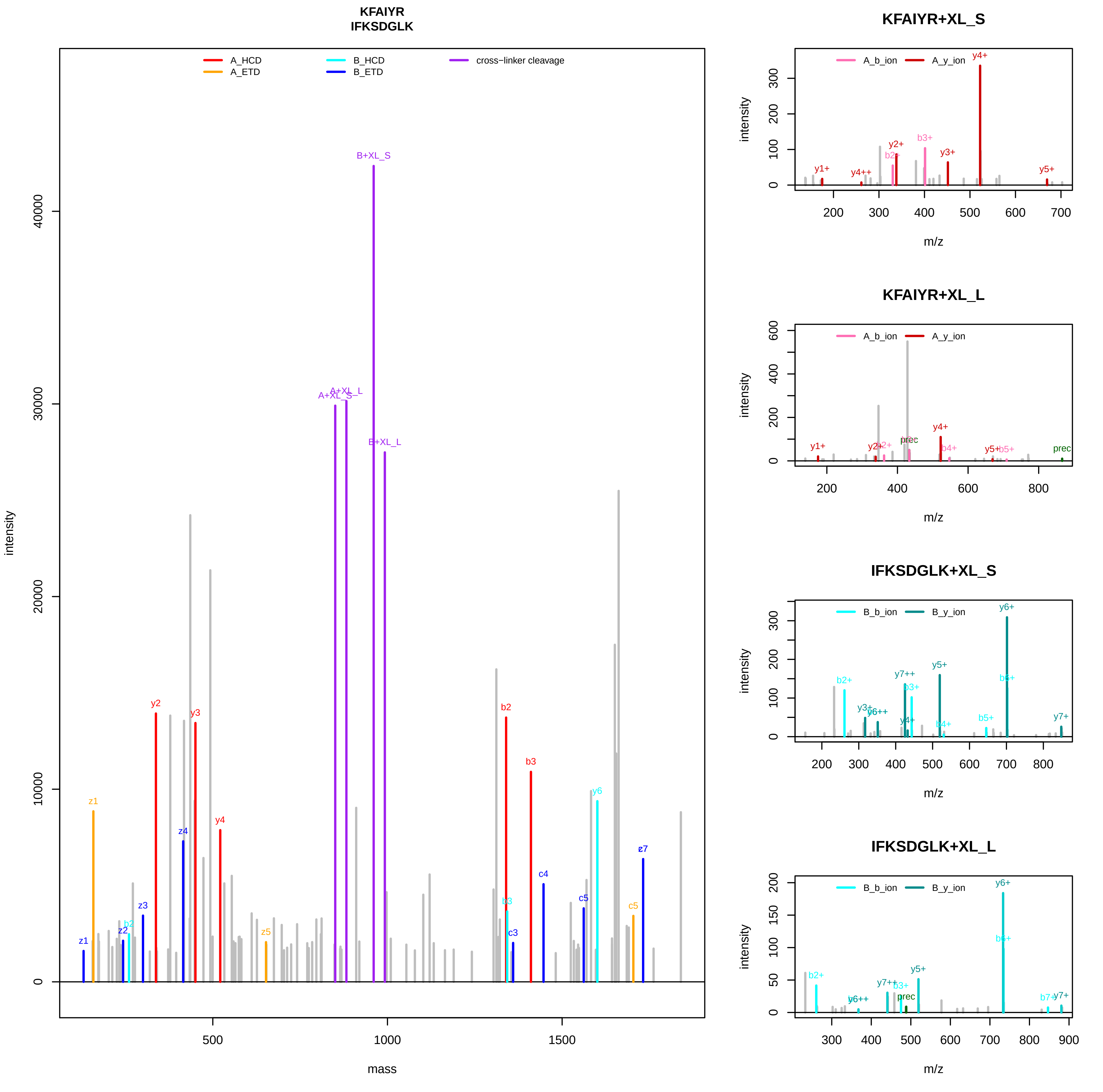
KFAIYR
IFKSDGLK
A_HCD
A_ETD
B_HCD
B_ETD
cross−linker cleavage
B+XL_S
A+XL_S
A+XL_L
B+XL_L
intensity
mass
z1
z2
b2
z3
y2
z4
y3
y4
z5
b2
b3
b3
c3
c4
c5
y6
z7
c5
KFAIYR+XL_S
A_b_ion
A_y_ion
y1+
y4++
b2+
y2+
b3+
y3+
y4+
y5+
m/z
KFAIYR+XL_L
prec
IFKSDGLK+XL_S
B_b_ion
B_y_ion
b2+
y3+
y6++
y7++
b3+
y4+
y5+
b4+
b5+
b6+
y6+
y7+
IFKSDGLK+XL_L
y7++
b3+
prec
b7+
